# Effects of Forming Parameters on Fatigue Life in Incremental Sheet Punching

**DOI:** 10.3390/ma14092308

**Published:** 2021-04-29

**Authors:** Jin Wang, Xu Wang, Yongqiang Wang, Mengting Wang

**Affiliations:** School of Mechanical and Automotive Engineering, Qingdao University of Technology, Qingdao 266525, China; wangxu523@126.com (X.W.); wyq199410042021@163.com (Y.W.); wangmengting618@163.com (M.W.)

**Keywords:** incremental sheet punching, fatigue comparative test, analysis of variance, Tukey test, *t*-test

## Abstract

In order to investigate the effects of main forming parameters on the fatigue life in incremental sheet punching, wavelength and amplitude were selected as factors, and fatigue life of truncated pyramids and virgin material was selected as indicator. The effects of the parameters were identified whereby the design of full factorial experiment, fatigue comparative test, analysis of variance, Tukey test, and *t*-test. It was found that wavelength and amplitude significantly affect the fatigue life. In addition, the improvement of fatigue life decreased with the increment of wavelength and increased with the increment of amplitude when it is less than a certain value, followed by decreasing.

## 1. Introduction

Incremental sheet forming (ISF) is a promising process technology for sheet metal in industry. ISF can complete the forming of parts at the ordinary Computerized Numerical Control (CNC) machine as well as special incremental forming machines with the help of the CAD model of the forming parts. Many advantages of ISF, e.g., without requirement for die or partial die, the cost is small and the forming cycle is short, flexibility for small batch sheet products, have been presented in detail by a lot of researchers. In recent years, this approach has been widely concerned and studied in the application fields of sheet metal products such as aviation, automobile, and medical treatment [1,2,3,4].

**Figure 1:**
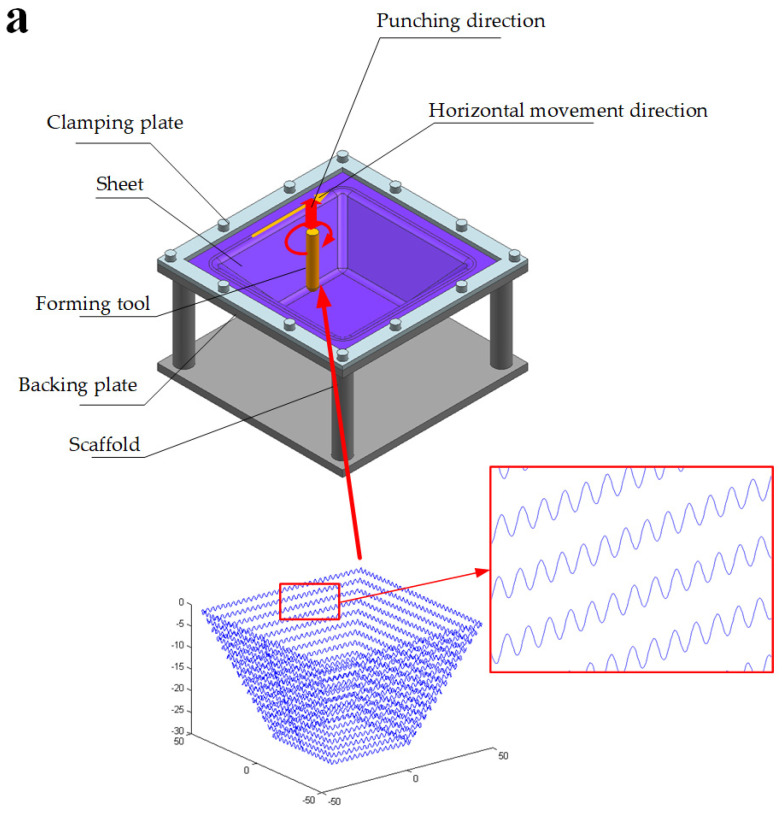

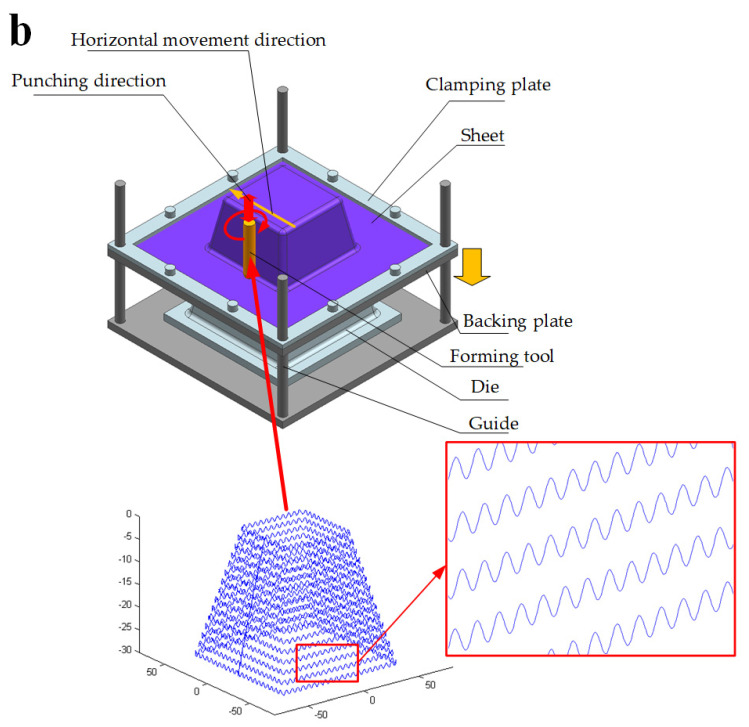
Schematic of incremental sheet punching driven by wave tool path. (**a**) single point forming; (**b**) two points forming.

Compared to conventional ISF, incremental sheet punching (ISP) of discontinued contact between forming tool and sheet metal in forming, has the advantages of higher sheet formability, lower forming force, and suitable for forming perforated sheet, which has attracted more attention in recent years. Schafe et al. [5] designed an ISP system by combining industrial robots with hammering devices. In this forming process, the tool path was controlled by the robot program, and the reciprocating motion in the direction perpendicular to the sheet metal was controlled by the high-frequency hammering device. Based on the system, Vihtonen [6] believed that the ISP can reduce the forming force and is applicability for forming perforated sheet. Luo et al. [7,8] developed a new ISP machine, based on the principle that the forming tool is fixed on the slider driven by the hydraulic cylinder, the tool driven by the hydraulic cylinder moves in the vertical direction of the sheet, and the horizontal movement of the sheet is controlled by the workbench. Sedighi et al. [9] designed an ISP mechanism camshaft, in which the cam mechanism was used to control the reciprocating motion owith af the forming tool perpendicular to the sheet, while the horizontal movement of the sheet was also controlled by the workbench. The calculation model of the pressure and velocity field of the ISP was derived by using the slip phenomenon and the upper bound method. Although ISP has many advantages, there are many problems such as low forming accuracy, long forming time, and the need for special equipment assistance, still need to be solved. Hence, multiple authors are seeking to select an reasonable process parameters to optimize ISP. ISP can reduce torsion and springback, as described by Schafer [5], who developed a method to generate the tool paths based on CAD model, using this method to form concave and convex parts.Wang et al. [10,11] proposed an approach, which can transform the tool paths of continuous contact in traditional ISF into sinusoidal wave tool paths perpendicular to the direction of sheet metal, as illustrated in Figure 1. And in this process ISP are allowed to achieved on ISF machine without adding any additional devices. Jiang et al. [12] studied the influence of main process parameters on forming time in incremental sheet punching driven by wave tool path using the design of experiment of orthogonal array, analysis of range, and analysis of variance. The results showed that the forming time is decreased with increasing tool vertical step, feed rate, wavelength, and tool diameter, but increased with increasing amplitude.

Fracture is one of the most important failure modes in mechanical parts and engineering components, since more than 80% of fracture accidents are caused by fatigue. Therefore, it is essential to experimentally evaluate the fatigue performance of ISF parts and introduce fatigue strength design to improve the fatigue life of parts in the design. She et al. [13] studied the residual stress of the truncated cone and found that the single-point incremental forming could improve the fatigue strength of sheet. Agrawal et al. [14] formed 90° bending parts by conventional bending, Single point incremental forming (SPIF), and the deformation machining process, and studied the effects of three processes on the fatigue life of parts through low cycles fatigue life test, which showed that SPIF parts had a longer fatigue life than those produced by deformation machining and conventional bending. Xu et al. [15] examine the fatigue life of parts formed employing accumulative-double-sided incremental forming (ADSIF) process, on AA2024-T3 sheets. The results indicate that the extrusion effect of the two tools on the both sides of the plate could lead to a more uniform distribution of residual compressive stress along the member wall, and therefor improving the fatigue life of the plate.

Although, related research on the fatigue performance of ISP is very rare, but it is necessary to understand the influence of wavelength and amplitude on fatigue life. Because the amplitude and wavelength were introduced into the ISP based on the common cutter path, and it was found that different combinations of wavelength and amplitude have obvious influence on the forming performance in the subsequent research, and it is also an important basis for the reasonable selection of process parameters in subsequent research.

In the given paper, based on the full factor experiment, ISP with different wavelengths and amplitudes was used to process the fixed parts, and then the fatigue comparison test is carried out on all fixed parts and unformed sheets [16].

## 2. Materials and Methods

### 2.1. Blanks Preparation

The material of plate used in this Experiment was 6061-T6 with 1 mm of thickness. Before forming, the sheets of 6061-T6 were cut into 150 mm × 150 mm blanks. The chemical compositions are shown in Table 1. The mechanical properties provided by the supplier (CHALCO Shandong Branch, Zibo, China) are listed in Table 2.

### 2.2. Incremental Sheet Punching

**Figure 2:**
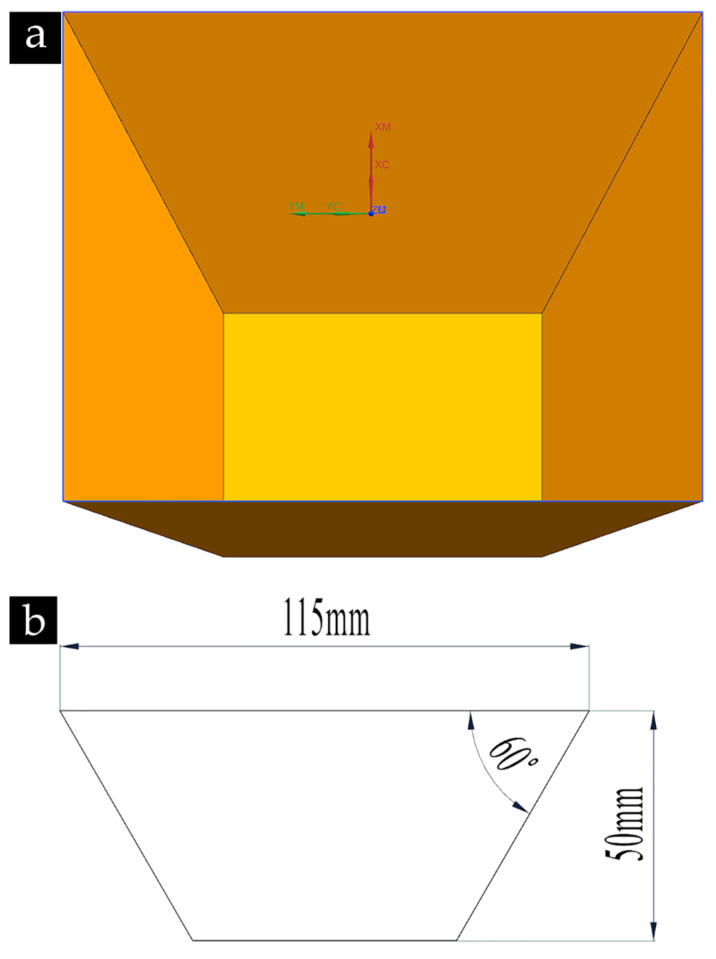
CAD model of the part (**a**) 3D model of the part; (**b**) section of the part.

In this experiment, truncated pyramids were formed by NHJ-1A ISF machine (designed and manufactured by Nanjing University of Aeronautics and Astronautics, Nanjing, China), the trun cated pyramids, shown as Figure 2. The sheets of 6061-T6 were fixed on the support of the NHJ-1A machine. The tool paths for continuous contact tool paths were generated by Siemens NX 12.0 (Siemens PLM Software, Munich, Germany) and converted the continuous contact tool paths to the wave paths through the method shown in [10]. The forming tool with a hemispherical end, made from high-speed steel, was selected. No.46 mechanical oil was used to reduce the coefficient of friction between the tool and the sheet. In order to investgate the influence of wavelength and amplitude on the fatigue life of sheet, based on the previous experience of ISF and ISP [10,11,12], the process parameters such as tool diameter, z-axis feed rate, feed speed, and hammering angle were set to constant values in this experiment, as shown in Table 3. Wavelength and amplitude were selected as factors to design the full-factor experimental scheme. The specific experimental scheme is given in Table 4.

### 2.3. Fatigue Comparative Test

**Figure 3:**
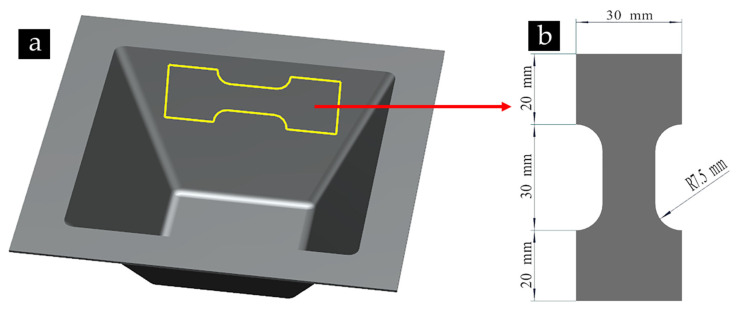
The shape and dimensions of fatigue test specimen. (**a**) specimen location; (**b**) fatigue test specimen.

To compare the effects of different wavelengths and amplitudes of ISP on the fatigue life of sheet, the fatigue contrast test was carried out on the high-frequency fatigue testing machine (PLG-100, Guanteng Automation, Changchun, China) under uniaxial stress-controlled tensile conditions at the same stress level [17]. The loading frequency of the testing machine is 100 Hz. To examine the fatigue resistance of the material under a relatively high-stress condition, the maximum stress was chosen as 180 MPa, which is 62% of the tensile strength of 6061-T6. The stress ratio was specified as R = 0.1. The test stops and automatically records the number of cycles when fatigue failure occurs under cyclic loading. The fatigue contrast test was divided into 9 experimental groups (truncated pyramids) and 1 treatment group (unprocessed 6060-T6 aluminum plate). The fatigue specimen is shown in Figure 3 [18], and the length direction of the specimen is the coincidence direction of the original fiber direction and the processing pattern of the aluminum alloy plate.

Given the confidence level 1 − α and the limit of relative error, the minimum number of specimens can be determined by the following relationship:

(1) $$n_{min}={\left(\frac{vt_{1-\alpha }}{\delta }\right)}^{2}={\left(\frac{s_{j}t_{1-\alpha }}{{\bar{x}}_{j}\delta }\right)}^{2}$$

where $n_{min}$ is minimum number of specimens, v is coefficient of variation, δ is Limit of relative error, $s_{j}$ and ${\bar{x}}_{j}$ are the standard deviation and average of subsample j, $t_{1-\alpha }$ can be found by t-distribution table.

In this study, the confidence level was set at 0.95, and δ was 5%. According to Equation (1), five specimens were selected for each group of samples [16].

### 2.4. Statistical Analysis

#### 2.4.1. ANOVA

ANOVA is a mathematical method that can quantitatively describe the statistical relationship between fatigue life and controllable factors based on experimental data [19]. It can be used to estimate the significance of each control factor on the overall response without the limitation of the number of comparison groups [20]. In this statistical analysis, the SPSS 25.0 (IBM, Armonk, NY, USA) was used to estimate the effects of two independent variables (wavelength and amplitude) on the overall fatigue life.

#### 2.4.2. Tukey Test

If the results of ANOVA reject the original assumption, indicating that the mean fatigue life corresponding to different parameters is not equal, and multiple comparisons are needed to further determine which two mean values are significantly different. In this paper, the Tukey test was used for multiple comparisons. Tukey test is mainly used for multiple comparisons with the same number of samples in three groups and more than three groups. An important advantage of the Tukey test is that it is very simple. As long as a value is calculated, all pairs of average differences can be compared. The test principle is illustrated as follows [21]:

Assume $\mu_{1}=\mu_{2}=\cdots =\mu_{k}=\mu$, so there are

(2) $$P\left(W\right)=P\left(\underset{1\leq j\leq m}{\max}\frac{{\bar{x}}_{j}-\mu }{\hat{\sigma }/\sqrt{n}}-\underset{1\leq k\leq m}{\min}\frac{{\bar{x}}_{k}-\mu }{\hat{\sigma }/\sqrt{n}}\geq \frac{c}{\hat{\sigma }/\sqrt{n}}\right)$$

let

(3) $$q\left(m,f_{e}\right)=\underset{1\leq j\leq m}{\max}\frac{{\bar{x}}_{j}-\mu }{\hat{\sigma }/\sqrt{n}}-\underset{1\leq k\leq m}{\min}\frac{{\bar{x}}_{k}-\mu }{\hat{\sigma }/\sqrt{n}}$$

where $q\left(m,f_{e}\right)$ is the distribution of studentized range with the freedom of m and $f_{e}$. When P ( W )=α, let

(4) $$c=q_{1-\alpha }\left(m,f_{e}\right)\hat{\sigma }/\sqrt{n}$$

where $q_{1-\alpha }\left(m,f_{e}\right)$ is the quantile of $q\left(m,f_{e}\right)$ at confidence level 1−α.

Comparing the size of $\left|{\bar{x}}_{j}-{\bar{x}}_{k}\right|$ and c, if $\left|{\bar{x}}_{j}-{\bar{x}}_{k}\right|\geq c$, it considers that there is a significant difference between the population means.

In this statistical analysis, this method is used to test the influence of two main effects (wavelength and amplitude) on fatigue life after ANOVA, and the influence degree of the main effect on fatigue life is estimated by confidence interval.

#### 2.4.3. *t*-Test

To understand whether the interaction between wavelength and amplitude has a significant effect on the fatigue life of the sheet, and to determine the degree of influence by using the confidence interval, it is necessary to make pairwise comparison between the experimental group and the treatment group. The *t*-test analysis method was used in this study. The principle of *t*-test is to use the theory of student distribution to deduce the probability of difference between two means. The *t*-test in this statistical analysis is mainly used to test the significance of the difference between the mean fatigue life of the experimental group and the treatment group, and to estimate the influence of the parameter interaction on the fatigue life of the sheet by the confidence interval. Estimates of impact by confidence intervals are as follows [16]:

(5) $$\left({\bar{x}}_{j}-{\bar{x}}_{k}\right)-t_{1-\alpha }s_{jk}\sqrt{\frac{1}{n_{j}}+\frac{1}{n_{k}}}<lg{\left[N^{*}\right]}_{j}-lg{\left[N^{*}\right]}_{k}<\left({\bar{x}}_{j}-{\bar{x}}_{k}\right)+t_{1-\alpha }s_{jk}\sqrt{\frac{1}{n_{j}}+\frac{1}{n_{k}}}$$

take anti-logarithm

(6) $$10^{\left({\bar{x}}_{j}-{\bar{x}}_{k}\right)-t_{\gamma }s_{jk}\sqrt{\frac{1}{n_{j}}+\frac{1}{n_{k}}}}\leq \frac{{\left[N^{*}\right]}_{j}}{{\left[N^{*}\right]}_{k}}\leq 10^{\left({\bar{x}}_{j}-{\bar{x}}_{k}\right)+t_{\gamma }s_{jk}\sqrt{\frac{1}{n_{j}}+\frac{1}{n_{k}}}}$$

where $s_{jk}$ is pooled standard deviation. ${\left[N^{*}\right]}_{j}$ and ${\left[N^{*}\right]}_{k}$ are the median fatigue life of two populations. For the given significance level α, the value of $t_{1-\alpha }$ can be obtained based on the t distribution tables.

In all the above analyses, significant level α was set at 5%, that is, confidence level (1−α ) was set at 95%.

## 3. Results and Discussion

### 3.1. Results of Fatigue Test

**Figure 4:**
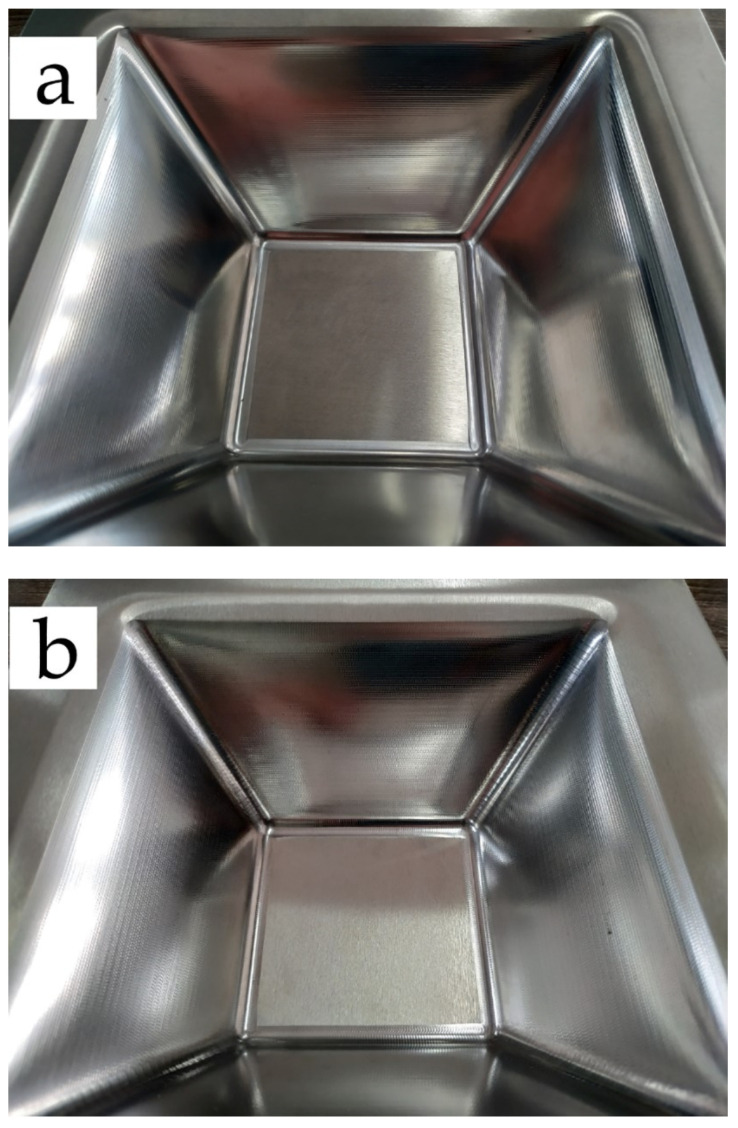

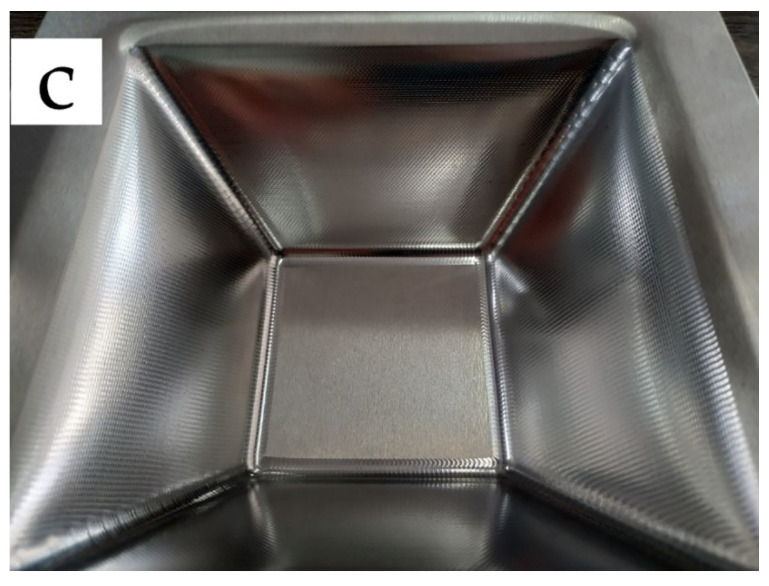
The formed parts in Table 5 (**a**) formed by wavelength 0.2 and amplitude 0.4; (**b**) formed by wavelength 0.6 and amplitude 0.4; (**c**) formed by wavelength 1.0 and amplitude 0.4.

**Figure 5:**
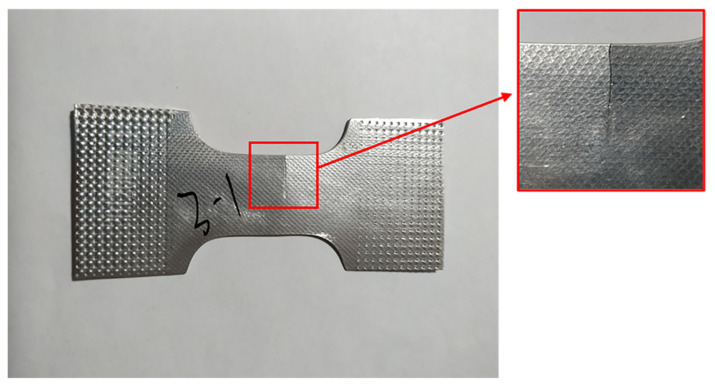
A specimen after fatigue test.

Partially formed parts and a fractured specimen after fatigue tests are shown in Figure 4 and Figure 5. Table 5 is the data measured by the group method, according to Table 5 the results of fatigue test are discrete under the same state. Therefore, effective statistics dealing with the large scale of fatigue data will be of great significance to obtain reasonable results. To facilitate observation and statistical analysis, the experimental data are assumed to be lognormal [22]. The logarithms of fatigue lives are shown in Table 6.

### 3.2. Statistical Analysis Results of Fatigue Contrast Test

#### 3.2.1. Test of Normality

Table 7 shows the statistical results of the normality test with different main effects (wavelength and amplitude). The values P of the wavelength, amplitude, and treatment group are more than 0.05, which means that the fatigue lives of the experimental group and the treatment group were in normal distribution.

#### 3.2.2. Levene Homogeneity of Variance Test

The homogeneity of variance test results are shown in Table 8. The value of $F_{1-\alpha }\left(s-1,\;n-s\right)$ can be obtained based on the F distribution tables. In the condition of this statistic experiment, the value of $F_{0.95}\;\left(9,\;40\right)$ is 2.12. The results show that the values W [23,24] are all less than 2.12, and the values P are more than 0.05, which means that the variance between samples is homogeneity.

#### 3.2.3. Results of ANOVA

Table 9 shows the statistical results of ANOVA with different main effects (wavelength and amplitude). The values P of the corrected model are less than 0.05, which means that at least one main factor has a significant effect on the fatigue life of sheet.

Numerous studies have shown that abilities of fatigue resistance of sample depend on its hardening depth and residual stress state [18]. Driven by wave tool path, ISP tool always imposes concentrated uniform axial forming force on the sheet, resulting in a fairly uniform distribution of compressive residual stresses along the component wall, which might explain the higher fatigue life observed for deformed material as compared to virgin material [15,25]. Moreover, due to the wave tool path, the surface strengthening effect similar to shot peening treatment will be produced by tool in the ISP, so that the residual tensile stress on the surface of the sheet is transformed into residual compressive stress, which inhibits the crack opening [26,27] and improves the fatigue life of sheet.

#### 3.2.4. Results of Tukey Test

**Figure 6:**
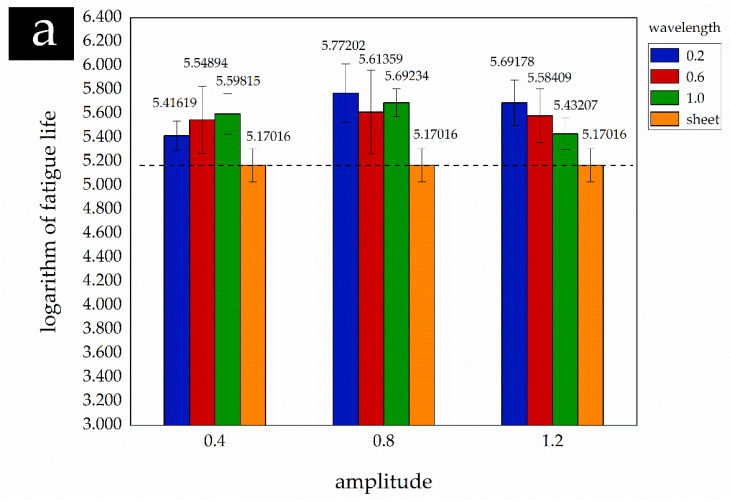

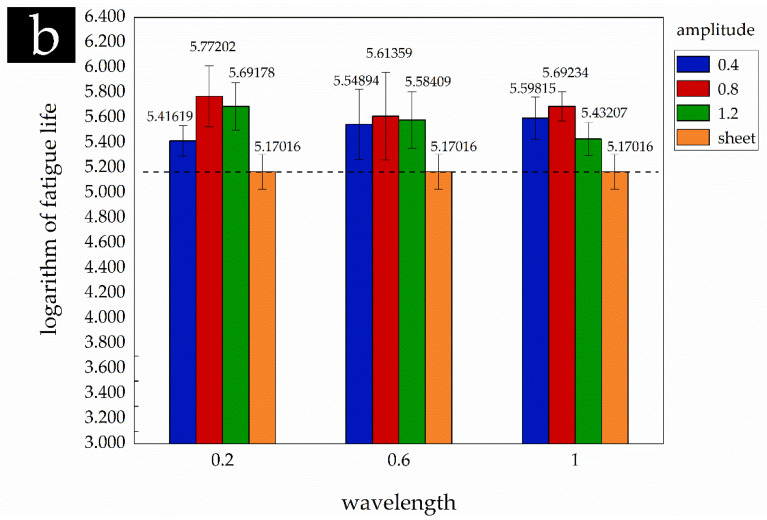
Comparison of mean fatigue life of sub-samples affected by different parameters. (**a**) affected by wavelength; (**b**) affected by amplitude.

**Figure 7:**
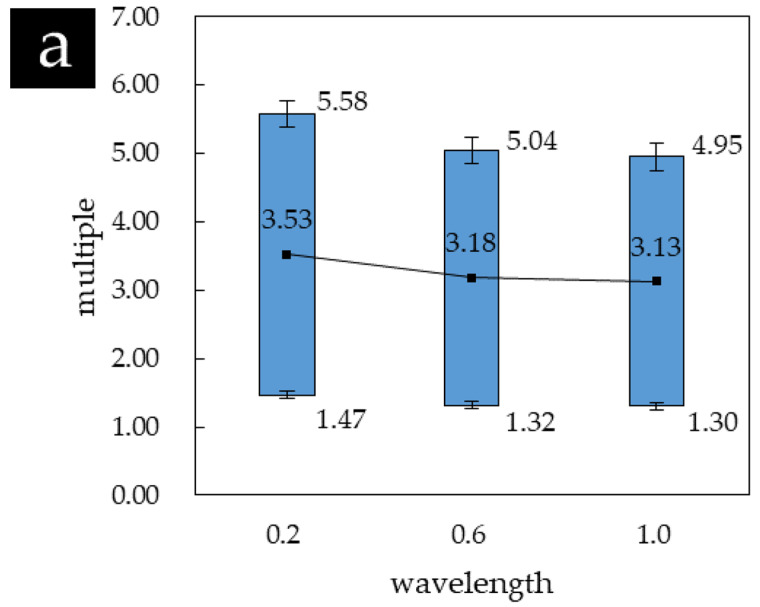

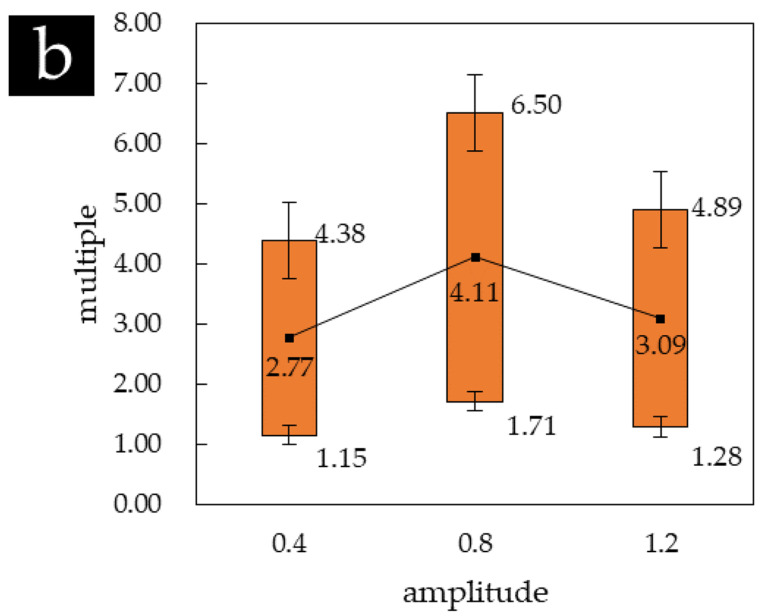
Comparison of creasing range of fatigue life by the main factor. (**a**) affected by wavelength; (**b**) affected by amplitude.

The statistical results of the Tukey test with different wavelengths are shown in Table 10. The results show that all values P are less than 0.05, which indicates that wavelength has obvious effects on fatigue life as compared to virgin material. Figure 6a shows the comparison results of the fatigue mean between the experimental group and the treatment group under the same amplitude. It is shown that the fatigue lives of the plate with different wavelengths have been significantly improved. Table 11 lists the improvement intervals of the fatigue life of the plate with different wavelengths, and the results are shown in Figure 7a. The trend in the figure indicates that higher values of wavelength lead to the lower improvement of fatigue life, and the maximum improvement of fatigue life was 1.47–5.58 times when wavelength equaled 0.2 mm.

It is well known that the fatigue life of sheet depends on the behaviors of crack initiation and propagation [18]. The uneven surface is easy to generate stress concentration to accelerate the initiation and propagation of fatigue cracks; thus. the roughness of specimen surface is one of the factors affecting the fatigue life. The increment of wavelength increases surface roughness [10], therefore, the improvement of fatigue life decreases with the increment of wavelength.

Table 12 shows the statistical results of the Tukey test with different amplitudes. Similar to the Tukey test results of the effect of wavelength on fatigue life, different amplitudes have significant effects on the fatigue life (p<0.05). Figure 6b shows that the mean fatigue lives with different amplitudes are significantly improved. The increased intervals of mean fatigue life are shown in Table 13, and the results are presented in Figure 7b. The results can be obtained that the improvement of fatigue life increases with the increment of amplitude when it is less than a certain value (it was equal to 0.8 mm in this paper), followed by decreasing, and the maximum improvement of fatigue life was 1.71–6.50 times.

In the process of shot peening strengthening, the projectile velocity is a factor affecting the magnitude of surface residual stress. The increment of projectile velocity shortens the impact interval time, and the residual compressive stress on the strengthened surface increases first and then decreases [28]. Therefore, the fatigue life shows the same trend as residual stress. Since the contact time between tool and plate increases with the increment of amplitude, similar to the shot peening strengthening, the influence of amplitude on fatigue life is the same as projectile velocity.

#### 3.2.5. Results of *t*-Test

**Figure 8:**
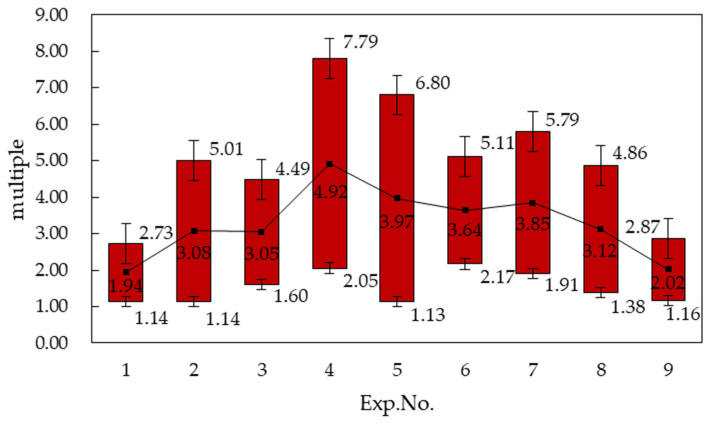
Comparison of creasing range of fatigue life by interaction.

The statistical results of the *t*-test by SPSS software are presented in Table 14 shows that values P were less than 0.05, which indicates that the interaction of parameters had a significant impact on the fatigue life of sheet. The influence area of the interaction of parameters on fatigue life can be estimated by the confidence interval, as shown in Table 15 and Figure 8, the results indicate that the maximum improvement of fatigue life with interaction was 2.05–7.79 times when wavelength equaled 0.2 mm and the amplitude equaled 0.8, which correspond to the main effect on fatigue life. Figure 8 also shows that the influence of wavelength and amplitude on the fatigue life of sheet metal is also applicable to the interaction of parameters.

Since ISP has the characteristics of processing complex and diverse parts, it has been commonly used in sheet metal forming fields such as automobile and aerospace [29]. The fatigue performance plays a decisive role in the failure of mechanical products, e.g.,the fatigue performance of the material of the aircraft fuselage affects the life accuracy of the aircraft structure and the life extension measures under the action of dynamic and static loads [22,30]. Therefore, evaluating the fatigue performance of ISP machining parts and introducing the fatigue strength design play a vitally important role in improving the fatigue life of parts. In this study, the influence of wavelength and amplitude on the fatigue performance of sheet was determined through the fatigue contrast test, and the optimum parameter combination for improving fatigue life was confirmed in a certain range. The test results may be worthwhile for determining the fatigue safety factor for the ISP parts’ design and manufacture.

## 4. Conclusions

Based on the small sample statistical analysis theory, the influence of different process parameters (wavelength and amplitude) of ISP on the fatigue life of 6061-T6 sheet material was studied by fatigue comparison test in this paper. The main conclusions are summarized as follows:

(1) ISP formed parts may have greater fatigue life under cyclic tension as compared to the virgin material. The improvement of fatigue life decreases with the increase of wavelength and increases with the increment of amplitude when it is less than a certain value, followed by decreasing.
(2) Under the given parameter values, the maximum improvement of fatigue life was 1.47–5.58 times when wavelength equaled 0.2 mm and was 1.71–6.50 times when amplitude equaled 0.8 mm.
(3) The maximum improvement of fatigue life with interaction was 2.05–7.79 times when wavelength equaled 0.2 mm and the amplitude equaled 0.8 mm.

In the future, the influence of different parameters on the fatigue limit of the plate will be studied by establishing the S–N curve of the parts by ISP, and the macro-mechanism and micro-mechanism of the impact of ISP on the fatigue life of the plate will be deeply understood by residual stress analysis and fracture analysis.

## Figures and Tables

**Table 1:** Chemical compositions of 6061 aluminum alloy sheet (%, mass fraction).

Mg	Si	Cu	Cr	Fe	Zn	Ti	Al
0.8–1.2	0.4–0.8	0.15–0.40	0.04–0.35	0.70	0.25	0.15	other

**Table 2:** Mechanical property parameters of 6061 aluminum alloy sheet.

Parameter	Value
Yield strength *R*_p0.2_/MPa	240
Tensile strength *R*_m_/MPa	290
Elongation *A*/%	6
Hardness /HBS	95
Elastic modulus *E*/GPa	68.3
Poisson ratio	0.33

**Table 3:** Experimental process parameters.

Tool Diameter (mm)	Vertical Feed (mm)	Feed Speed (mm/min)	Hammer Angle (°)
10	0.5	1000	90

**Table 4:** Full factorial experiment plan.

No.	Factor	
	Wavelength(mm)	Amplitude(mm)
1	0.2	0.4
2	0.6	0.4
3	1.0	0.4
4	0.2	0.8
5	0.6	0.8
6	1.0	0.8
7	0.2	1.2
8	0.6	1.2
9	1.0	1.2

**Table 5:** Results of fatigue life test.

No.	Cycle of Fatigue Life (×10^5^)				
1	1.897	2.019	2.804	3.053	3.675
2	1.547	2.732	2.964	5.510	8.048
3	2.472	3.311	3.556	5.030	6.687
4	3.504	4.096	4.323	9.476	12.325
5	1.240	3.940	4.144	4.801	12.030
6	3.793	3.970	4.495	6.367	6.718
7	2.730	3.740	5.249	6.758	7.943
8	2.384	2.504	3.629	4.489	8.562
9	1.915	2.380	2.535	2.918	4.291
10 ^a^	0.893	1.435	1.610	1.611	2.134

^a.^ treatment group.

**Table 6:** Logarithm of fatigue life.

No.	$\mathrm{Logarithm}\;\mathrm{of}\;\mathrm{Fatigue}\;\mathrm{Life}\;\;x_{ji}=lgN_{ji}$										*p*(%)	*μ_pi_*
	$x_{1i}\;$	$x_{2i}$	$x_{3i}$	$x_{4i}$	$x_{5i}$	$x_{6i}$	$x_{7i}$	$x_{8i}$	$x_{9i}$	$x_{10i}{\;}^{a}$		
1	5.278	5.189	5.393	5.545	5.093	5.579	5.436	5.377	5.282	4.951	83.33	−0.9685
2	5.305	5.436	5.520	5.612	5.595	5.599	5.573	5.399	5.377	5.157	66.67	−0.4313
3	5.448	5.472	5.551	5.636	5.617	5.653	5.720	5.560	5.404	5.207	50.00	0.0000
4	5.485	5.741	5.702	5.977	5.681	5.804	5.830	5.652	5.465	5.207	33.33	0.4313
5	5.565	5.906	5.825	6.091	6.080	5.827	5.900	5.933	5.633	5.329	16.67	0.9685

^a.^ treatment group; p: maternal survival rate; $u_{pi}$: normal deviation.

**Table 7:** Normality test results of main effect fatigue life.

Source	Level	r	df	p
Wavelength	0.2	0.958	15	0.666
	0.6	0.984	15	0.989
	1.0	0.957	15	0.646
Amplitude	0.4	0.969	15	0.849
	0.8	0.889	15	0.065
	1.2	0.929	15	0.264
Treatment group	Sheet	0.911	5	0.473

**Table 8:** Test results of homogeneity of variance.

Source	Based on	W	df1	df2	p
Logarithm of Fatigue life	Mean	1.011	9	40	0.448
	Median	0.588	9	40	0.799
	trimmed mean	0.990	9	40	0.463

df1: Freedom of molecules; df2: Freedom of denominator.

**Table 9:** Results of ANOVA.

Source	Type III Sum of Squares	df	Mean Square	F	p
Corrected Model	1.366 ^a^	9	0.152	3.448	0.003
Wavelength	0.024	2	0.012	0.272	0.763
Amplitude	0.235	2	0.117	2.667	0.082
Wavelength × amplitude	0.298	4	0.074	1.690	0.171
Error	1.761	40	0.044		
Total	1544.326	50			
Corrected Total	3.127	49			

^a.^ R Squared = 0.437 (Adjusted R Squared = 0.310).

**Table 10:** Effect of wavelength on fatigue life.

(I) Wavelength	(J) Treatment Group	Mean Difference (I − J)	Std. Error	p	95% Confidence Interval	
					Lower Bound	Upper Bound
0.2	Sheet	0.45650 *	0.108344	0.001	0.16609	0.74691
0.6		0.41204 *	0.108344	0.003	0.12163	0.70245
1.0		0.40402 *	0.108344	0.003	0.11362	0.69443

Based on observed means.The error term is Mean Square (Error) = 0.044. * The mean difference is significant at the 0.05 level.

**Table 11:** Increasing range of fatigue life by wavelength.

Treatment Group	Wavelength	Multiple
Sheet	0.2	1.47–5.58
	0.6	1.32–5.04
	1.0	1.30–4.95

**Table 12:** Effect of amplitude on fatigue life.

(I) Amplitude	(J) Treatment Group	Mean Difference (I − J)	Std. Error	p	95% Confidence Interval	
					Lower Bound	Upper Bound
0.4	Sheet	0.35093 *	0.108344	0.012	0.06052	0.64134
0.8		0.52249 *	0.108344	0.000	0.23208	0.81289
1.2		0.39915 *	0.108344	0.004	0.10874	0.68956

Based on observed means. The error term is Mean Square(Error) = 0.044. *. The mean difference is significant at the 0.05 level.

**Table 13:** Increasing range of fatigue life by amplitude.

Treatment Group	Amplitude	Multiple
Sheet	0.4	1.15–4.38
-	0.8	1.71–6.50
-	1.2	1.28–4.89

**Table 14:** Results of *t*-test.

Treatment Group	Experimental Group	t	p	95% Confidence Interval of the Difference	
				Lower	Upper
Sheet	1	2.988	0.017	0.056151	0.435908
-	2	2.718	0.026	0.057470	0.700078
-	3	4.403	0.002	0.203855	0.652125
-	4	4.792	0.001	0.312213	0.891508
-	5	2.628	0.030	0.054273	0.832573
	6	6.474	0.000	0.336187	0.708156
-	7	4.985	0.001	0.280337	0.762901
-	8	3.498	0.008	0.141032	0.686822
-	9	3.088	0.015	0.066308	0.457512

**Table 15:** Comparison of creasing range of fatigue life by interaction.

Treatment Group	Experimental Group	Multiple
Sheet	1	1.14–2.73
-	2	1.14–5.01
-	3	1.60–4.49
-	4	2.05–7.79
-	5	1.13–6.80
-	6	2.17–5.11
-	7	1.91–5.79
-	8	1.38–4.86
-	9	1.16–2.87

## Data Availability

The data presented in this study are available in this article.
